# ASSOCIATION BETWEEN PRE–HIP FRACTURE ANTIDEPRESSANT USE AND HOSPITAL LENGTH OF STAY AMONG MEDICARE BENEFICIARIES

**DOI:** 10.1093/geroni/igae098.0521

**Published:** 2024-12-31

**Authors:** Rhea Mehta, Jason Falvey, Chixiang Chen, Yu Dong, Michelle Shardell, Takashi Yamashita, Denise Orwig

**Affiliations:** University of Maryland Baltimore, Baltimore, Maryland, United States; University of Maryland School of Medicine, Baltimore, Maryland, United States; University of Maryland School of Medicine, Baltimore, Maryland, United States; University of Maryland School of Medicine, Baltimore, Maryland, United States; University of Maryland School of Medicine, Baltimore, Maryland, United States; University of Maryland Baltimore, Baltimore, Maryland, United States; University of Maryland School of Medicine, Baltimore, Maryland, United States

## Abstract

Antidepressants are the first-line treatment for depression among older adults. Their use presents important sex differences and is linked to hospital length of stay (LOS), which can affect hip fracture recovery. Given the push to deprescribe these medications among older adults, exploring the effect of antidepressant use prior to hip fracture among older adults with existing depression is important. Thus, our study examined the association between pre-fracture antidepressant use and hospital LOS among hip fracture survivors, and related sex differences. The sample included 17,936 community-dwelling Medicare fee-for-service beneficiaries with depression and a hospitalization claim for hip fracture surgery between 2010 and 2017. Ordinal logistic regression was used to estimate the association between pre-fracture antidepressant use, measured 6 months prior to fracture, and hospital LOS in days, a categorical outcome variable classified into three groups (1-4, 5-8, and 8+ days) during the 30-day post-fracture period. In the covariate-adjusted model, hip fracture survivors with depression who used antidepressants pre-fracture had 6.7% higher odds of a shorter hospital LOS compared to non-users (odds ratio [OR]=1.07; 95% CI=1.01, 1.13; p=0.03). Males had longer LOS on average (mean=6.02, standard deviation [SD]=4.07) compared to females (mean=5.43, SD=3.17), but the sex-by-antidepressant use interaction was not significant (p=0.83). Treating existing depression with antidepressants was associated with shorter hospital LOS in this sample. The findings suggest a benefit of antidepressant use prior to hip fracture which may inform clinical decision-making surrounding depression management among hip fracture survivors to optimize recovery trajectories.

